# Efficacy of *Vitex pseudo‐negundo* in a Bovine Mastitis Model Experimentally Induced by *Staphylococcus aureus* in Rats

**DOI:** 10.1002/vms3.71031

**Published:** 2026-06-16

**Authors:** Hüseyin Koç, Arzu Fındık, Tolga Güvenç, Nevzat Batan, Seda Fandaklı, Murat Fındık

**Affiliations:** ^1^ Department of Veterinary Medicine, Vocational School of Maçka Karadeniz Technical University Trabzon Türkiye; ^2^ Department of Veterinary Microbiology, Faculty of Veterinary Medicine Ondokuz Mayıs University Samsun Türkiye; ^3^ Department of Veterinary Pathology, Faculty of Veterinary Medicine Ondokuz Mayıs University Samsun Türkiye; ^4^ Department of Molecular Biology and Genetics, Faculty of Science Karadeniz Technical University Trabzon Türkiye; ^5^ Department of Medical Services and Techniques, Vocational School of Health Services Artvin Coruh University Artvin Türkiye; ^6^ Department of Veterinary Obstetrics and Gynecology, Faculty of Veterinary Medicine Ondokuz Mayıs University Samsun Türkiye

**Keywords:** bacterial load, experimental model, mastitis, oxidative stress, phytotherapy, *Vitex pseudo‐negundo*

## Abstract

**Background:**

Mastitis is one of the most prevalent and economically significant diseases in dairy cattle, causing to inflammation of the mammary gland and reduced milk yield.

**Objectives:**

This study investigated the therapeutic efficacy of *Vitex pseudo‐negundo* (VPN) extracts in an experimentally induced rat mastitis model.

**Methods:**

Sixty female Wistar rats were randomly assigned into six groups of 10. Group I served as the control group. Mastitis was induced by intramammary injection of *Staphylococcus aureus* (SA) (1.5 × 10^8^ cfu/mL; 10 µL per gland) into the L4, L5, R4 and R5 mammary glands. Group III received intramammary gentamicin (0.25 mg/10 µL/day) for 5 days. Groups IV–VI received VPN extracts—water (0.2603 µg/kg/day), ethanol (0.0803 µg/kg/day) and petroleum ether (0.1042 µg/kg/day), administered at 10 µL per gland for the same duration. Rats were clinically monitored three times daily from the onset of clinical signs until euthanasia.

**Result:**

SA infection significantly increased mammary gland weight, total oxidative status (TOS) and bacterial load and induced tissue pathology, while decreasing total antioxidant status (TAS). C‐reactive protein levels remained unchanged. VPN significantly improved mammary gland weight, TOS, TAS, bacterial load and histological parameters (*p* < 0.001). Mammary gland weight, oxidative stress, bacterial load and histopathological lesion severity all decreased markedly following treatment. Histological examination showed near‐normal restoration of mammary gland architecture in all treatment groups.

**Conclusion:**

Preparations of VPN, particularly the water extract, demonstrated strong potential for restoring altered microbiological, biochemical and histological parameters in this rat mastitis model.

## Graphical abstract

This study evaluated the therapeutic efficacy of *Vitex pseudo‐negundo* (VPN) extracts in a *Staphylococcus aureus*‐induced mastitis rat model. Following extraction with water, methanol and petroleum ether, in vitro MICs were determined. Rats established with mastitis received 5‐day treatments. Although serum CRP showed no significant changes, mammary tissue analyses revealed that VPN extracts, particularly the water extract, significantly reduced bacterial load, improved histopathological architecture and ameliorated oxidative stress markers (TAS/TOS), demonstrating therapeutic efficacy comparable to the standard gentamicin treatment.

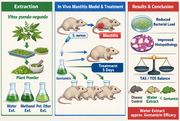

## 1 Introduction

Dairy farming plays a critical role in meeting the global demand for animal protein (Akman et al. 2010). Increasing urbanization and food consumption have heightened the need for efficient, high‐quality milk production (Medeiros et al. 2022). Mastitis, a major problem affecting milk production, causes substantial economic losses and poses public health risks (Antók et al. 2019; Cobirka et al. 2020). The disease is characterized by elevated somatic cell counts, which directly affect milk quality and safety (Ruegg and Pantoja 2013). Antibiotics commonly used to treat mastitis can leave residues in dairy products, contribute to antibiotic resistance and promote the spread of zoonotic pathogens (Cheng and Han 2020; Kyuchukova 2020). Furthermore, the potential transmission of methicillin‐resistant *Staphylococcus aureus* (SA) from animals to humans highlights the public health importance of mastitis (García‐Álvarez et al. 2011; Li and Zhao 2018).

Consequently, the pursuit of secure and efficacious herbal alternatives to antibiotic use is gaining paramount significance (Barlow 2011; Feudis and Drieu 2004). From this perspective, *Vitex pseudo‐negundo* (VPN), assessed in this study, is a viable option given its historical use and phytochemical profile. VPN is a flowering plant from the Verbenaceae family, naturally occurring in Turkey's flora (Davis 1982; Tübives 2024).

VPN comprises primary phytochemical components such as flavonoids (e.g., vitexin, isovitexin and luteolin), iridoid glycosides, diterpenes, phenolic compounds and essential oils (Almuslih and Al‐Assie 2020). These compounds have been documented in scientific literature for their antibacterial, anti‐inflammatory and antioxidant activities. Flavonoids are recognized for their therapeutic potential in infectious disorders owing to their ability to scavenge free radicals and suppress the release of inflammatory cytokines.

In in vitro investigations of VPN, the plant has shown considerable antibacterial efficacy against SA, the primary pathogen responsible for mastitis (Almuslih and Al‐Assie 2020). The investigation revealed that the antibacterial efficacy of VPN surpassed that of the *Vitex agnus‐castus* (VAC) species within the same family. The absence of in vivo research assessing VPN in animal models is a significant gap in the literature.

Consequently, VPN was selected for the mastitis model in rats because of its antibacterial properties, extensive phytochemical composition and the absence of prior testing in animal mastitis models. Regarded as a phytotherapeutic substance, VPN may offer a non‐antibiotic alternative for mastitis therapy. Therefore, the present study aimed to investigate the in vivo efficacy of VPN extracts in an experimentally induced rat mastitis model. This study was designed on the basis of the hypothesis that VPN may mitigate inflammatory cell accumulation in mammary tissue, which is associated with somatic cell proliferation during mastitis.

### 1.1 Collection, Identification and Drying of Plants

Specimens of VPN were meticulously collected with minimal ecological disruption, ensuring full anatomical features (leaf, flower and fruit) were preserved. Habitat characteristics, environmental data and geographical information were recorded. Taxonomic identification was performed through macroscopic and microscopic evaluations, supported by standard botanical references (Bramley et al. 2009; Cortini Pedrotti 2001; Davis 1982; Frey et al. 2006). Samples were dried under controlled conditions, avoiding direct sunlight to maintain structural and chemical integrity (Elibo et al. 2011). Dried specimens were stored under stable and dry conditions to preserve their properties for extraction and analysis.

### 1.2 Preparation of Plant Extracts

The aerial parts were powdered and extracted via maceration using water, methanol and petroleum ether solvents. Each extraction was repeated three times for 24 h at room temperature. After filtration, solvent removal was performed under vacuum below 40°C to preserve thermolabile compounds. The resultant crude extracts were used in subsequent biological assays (Asdadi et al. 2015; Çalış et al. 1985; Zareshahrabadi et al. 2023).

### 1.3 Determination of Minimum Inhibitory Concentration (MIC) Value of Plant Extracts

The antibacterial activity of the extracts against SA was evaluated using the tube dilution method (Arda 2000). Serial dilutions were prepared, inoculated with bacterial suspension, incubated at 37°C for 24 h and visually assessed for bacterial growth inhibition. The MIC was determined as the lowest concentration without visible growth (Arda 2000). These values were evaluated in conjunction with the applied volume, and the dose calculation in µg/kg was made for each extract. The volume used in the in vivo application was 10 µL, and the doses calculated for 300 g rats were, respectively, 0.2603, 0.0803 and 0.1042 µg/kg.

### 1.4 Establishment of Experimental Groups

Sixty female Wistar rats (12–16 weeks old, 280–320 g) were randomized into six groups (*n* = 10 each):

The number of animals per group (*n* = 10) was determined on the basis of published experimental mastitis models reporting reliable biochemical and histological outcomes using 8–12 animals per group (Taifa et al. 2022). A priori power analysis was conducted using GPower, adopting *α* = 0.10 and power (1 − *β*) = 0.70, parameters commonly accepted in exploratory pharmacodynamic and preclinical herbal studies (Brysbaert 2019; Bujang 2021; Serdar et al. 2021; Wulff and Taylor 2024). Similar alpha and power levels have been applied in multiple Phase II preclinical investigations (Hosoda et al. 2019; Okabe et al. 2022; Takahashi et al. 2023), ensuring a balance between statistical robustness and ethical animal use.

To differentiate the component‐specific effects of the plant, three extraction systems (aqueous, ethanol and petroleum ether) were prepared. Before intramammary administration, all organic solvents were completely removed under vacuum, and sterile physiological saline was used solely as the carrier. Therefore, the control group received physiological saline only, which is a standard and biocompatible vehicle in mammary administration studies.

The safety of physiological saline on mammary tissue was verified through the absence of erythema, oedema or pain upon inspection; preserved epithelial and stromal architecture without inflammatory infiltration in histology; bacterial load values consistent with healthy controls; and low total oxidative status (TOS) and high total antioxidant status (TAS) values, indicating no oxidative stress.

Additionally, a healthy non‐infected negative control group was included to confirm that physiological saline did not induce any degenerative or inflammatory changes in mammary tissue.

Group I (control): Received intramammary injection of physiological saline.

Group II (illness): Infected intramammarily with SA and euthanized after 48 h.

Group III (gentamicin): Infected and treated intramammarily with gentamicin (0.25 mg/10 µL) once daily for 5 days. The 5‐day treatment duration for all groups was determined in accordance with previously published protocols of experimental mastitis in rats (Taifa et al. 2022).

Groups IV–VI (VPN Extracts): Infected and treated with aqueous, ethanol or petroleum ether extracts (10 µL per mammary gland) for 5 days.

Rats were maintained under standard laboratory conditions (20–22°C, 45%–65% humidity, 12 h light/12 h dark cycle) with ad libitum access to food and water.

### 1.5 Induction of Mastitis by *Staphylococcus aureus* in Rats

SA strains isolated from bovine mastitis cases (Ondokuz Mayıs University) were cultured, standardized to 1.5 × 10^8^ cfu/mL (0.5 McFarland standard) and injected intramammarily (10 µL) into the L4, L5, R4 and R5 mammary glands under anaesthesia. SA was isolated and prepared following the method described by Taifa et al. (2022). Control animals received sterile physiological saline injections (Taifa et al. 2022).

In our study, intramammary injections were performed through the teat canal using a 30G fine needle under a surgical microscope to ensure precise canal cannulation and to minimize mechanical trauma. All administrations were conducted under aseptic conditions by a single experienced operator, with an injection volume of 10 µL per mammary gland, thereby avoiding intraductal pressure elevation or tissue stretching. Throughout the experimental period, no macroscopic signs of irritation, such as erythema, oedema or discharge, were observed.

Furthermore, histopathological examination of control mammary tissues revealed no epithelial laceration, transmural haemorrhage or iatrogenic damage, indicating that the procedure did not induce trauma. These histological findings were consistent with the oxidative balance parameters, as the control group exhibited low TOS and high TAS levels, confirming that neither the carrier (physiological saline) nor the injection technique caused oxidative stress or tissue injury.

Regarding the rationale for using rats, this model was chosen because it represents a cost‐effective, ethically compliant and reproducible system for evaluating mastitis pathogenesis and therapeutic efficacy in vivo. The rat mammary gland shares anatomical and histological similarities with bovine mammary tissue, allowing for realistic simulation of infection dynamics, controlled intramammary inoculation, serial sampling and detailed histopathological evaluation. As reported in the literature, the rat mastitis model provides a reliable framework for investigating acute and subacute inflammatory processes, bacterial colonization and oxidative stress modulation (Taifa et al. 2022). Hence, it was adopted in accordance with the 3R (Replacement, Reduction, Refinement) principles to ensure both scientific validity and ethical soundness.

### 1.6 Measurements of Mammary Gland Weight

The weights of the mammary glands were measured using a precision scale after the animals were sacrificed under general anaesthesia and exsanguinated after the procedures were completed for each group.

### 1.7 Biochemical Measurements

After the mastitis model was created on the experimental animals and the necessary experimental procedure was completed for each group, they were sacrificed under general anaesthesia by the bloodletting method, their blood was taken and placed in gel serum separation tubes, and C‐reactive protein (CRP) measurements were performed on the same day using the vc‐CRP‐P kit and DL (CRP) diluent with a Fujifilm DRI‐CHEM NX500I brand and 96364505 serial number device.

### 1.8 Bacterial Load Measurements

Mammary tissues were homogenized aseptically in physiological saline, serially diluted and plated onto Tryptic Soy Agar (TSA). After 24 h of incubation at 37°C, colony‐forming units (cfu) were counted, and results were expressed as log_10_ cfu/g of tissue (Benson 1973). The bacterial load was quantified as cfu/g of breast tissue (cfu/g) and represented on a logarithmic scale as log_10_ cfu/g. The below formula was employed for the quantification of bacterial load:

Bacterial load (cfu/g) = number of visible colonies on the plate × dilution factor. This approach was utilized to conduct a comparative assessment of bacterial colonization among experimental groups and to objectively ascertain the microbiological impacts of mastitis (Benson 1973).

### 1.9 Oxidative Stress Measurements

R4 mammary gland homogenate was assessed for TOS and TAS according to standard procedure (Erel 2004, 2005). For this, Elabscience (TAS Cat. No.: E‐BC‐K801‐M) and (TOS Cat. No.: E‐BC‐K802‐M) kits were used.

### 1.10 Histopathological Examinations

R5 mammary glands were fixed in 10% formaldehyde, dehydrated, embedded in paraffin and sectioned at 5 µm thickness. Haematoxylin–eosin (H&E) staining was performed. Histological evaluations included inflammation, epithelial integrity, glandular atrophy, vascular changes and fibrosis, scored semi‐quantitatively (0–3 scale). Microscopic examinations were performed using Nikon E‐400 and images were captured with Nikon DS‐L2 5M camera (Suresh et al. 2022; Taifa et al. 2022). Somatic cell accumulation was indirectly evaluated by histological scoring of inflammatory cell infiltration in mammary tissue sections stained with H&E, following the approach described by Antók et al. (2019).

### 1.11 Statistical Evaluation

Statistical analysis of the data was performed using the ANOVA test with the SPSS 23.0 package programme (SPSS Inc., Chicago, IL). Data were tested for normality using the Shapiro–Wilk test and were found to be normally distributed (*p* > 0.05). Therefore, one‐way ANOVA followed by Tukey's post hoc test was used to evaluate intergroup differences. Statistical significance was defined as *p* < 0.05. The interpretation of correlation strength followed standard classification guidelines (Dancey and Reidy 2007; Schober et al. 2018), *r* = 0.00–0.19 very weak, 0.20–0.39 weak, 0.40–0.59 moderate, 0.60–0.79 strong and 0.80–1.00 very strong. These criteria were used to describe correlation results in the manuscript. Correlation analyses were conducted using the Pearson correlation coefficient (*r*) to determine the relationships among biochemical, bacteriological and histopathological variables, with significance accepted at *p* < 0.05.

## 2 Results

### 2.1 Clinical Results

Clinical signs of mastitis were closely monitored at 6, 12, 24 and 48 h following intramammary SA injection. Initial inflammatory responses, such as erythema, oedema, localized heat and swelling of the mammary gland, were observed early after infection. Symptom severity increased over time, culminating in severe oedema and purulent exudate formation by 48 h. Infected Groups (II–VI) displayed pronounced inflammation starting at 24 h, peaking at 48 h post‐inoculation. Upon treatment initiation, Groups IV–VI, which received VPN extracts, exhibited a gradual regression of clinical signs. Notable improvement was evident by 48 h post‐treatment, and by the fifth day, most symptoms had resolved. Gentamicin‐treated animals (Group III) demonstrated faster recovery, with significant symptom reduction within 24–48 h and near‐complete healing by the third day. By the fifth day, mammary tissues had largely returned to normal appearance. Overall, the water extract of VPN showed a therapeutic effect comparable to or exceeding that of gentamicin in resolving clinical symptoms, supporting the potential of phytotherapeutic approaches in mastitis management.

### 2.2 Mammary Gland Weight Measurement

**FIGURE 1:**
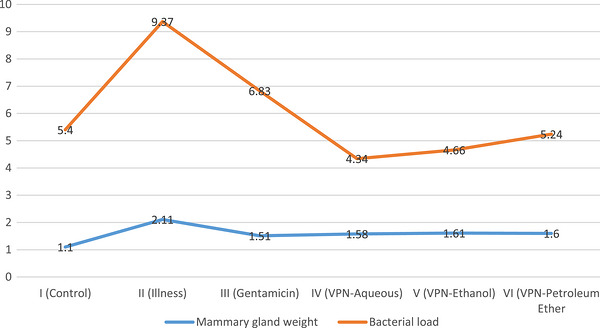
Mammary gland weight: Mammary gland weight was significantly increased in the disease group (Group II) and reduced in all treatment groups (*p* < 0.000). No significant differences were observed between gentamicin and extract‐treated groups, nor among the extract groups themselves (*p* > 0.05). Bacterial load: Bacterial load was highest in Group II and significantly reduced in all treatment groups (*p* < 0.000). Group IV showed the lowest load among all groups. No significant differences were observed between Groups IV and V or between Group VI and Group I (*p* > 0.05). VPN, *Vitex pseudo‐negundo*.

In rats with an experimental mastitis model, the left fourth (L4) mammary glands were dissected and quantitatively measured to determine mammary gland weight. Mean mammary gland weights (g): Group I (control): 1.10 ± 0.03 g, Group II (disease): 2.11 ± 0.04 (g), Group III (gentamicin‐treated group): 1.51 ± 0.03 g, Group IV (water extract‐treated group): 1.58 ± 0.01 g, Group V (ethanol extract‐treated group): 1.61 ± 0.02 g and Group VI (petroleum ether extract‐treated group): 1.60 ± 0.04 g (Figure 1).

The mammary gland weight in Group II (disease group) was significantly higher compared to all other groups (*p* < 0.001). When evaluated among the treatment groups, mammary gland weight was significantly lower in Group III (gentamicin‐treated group) compared to infected but untreated Group II (*p* < 0.001). Mammary gland weights were significantly lower in the herbal extract‐treated groups (Groups IV, V and VI) compared to Group II (*p* < 0.001); however, the differences between the herbal extract‐treated groups (Groups IV, V and VI) and gentamicin‐treated Group III were not statistically significant (*p* > 0.05). Furthermore, there was no statistically significant difference in mammary gland weights between Group IV (water extract), Group V (ethanol extract) and Group VI (petroleum ether extract) (*p* > 0.05).

### 2.3 Biochemical Measurements

CRP levels, indicative of systemic inflammation, were assessed in 60 rats using the Fujifilm DRI‐CHEM NX500I analyser. All animals exhibited CRP levels below 0.3 mg/dL. Statistical analysis confirmed no significant differences between groups.

### 2.4 Bacterial Load Measurements

Bacterial loads were determined by homogenizing L5 mammary glands and calculating log_10_ cfu/gr values. Mean bacterial loads were Group I (control): 5.40 ± 0.10, Group II (disease): 9.37 ± 0.06, Group III (gentamicin): 6.83 ± 0.15, Group IV (water extract): 4.34 ± 0.09, Group V (ethanol extract): 4.66 ± 0.16 and Group VI (petroleum ether extract): 5.24 ± 0.31 (Figure 1).

The bacterial load in Group II was significantly higher compared to Groups I, III, IV, V and VI (*p* < 0.001). There was no statistically significant difference in bacterial loads between Groups I and VI (*p* > 0.05). However, the bacterial load reduction capacity of Group VI was similar to that of Group III. The bacterial load detected in Group III showed a statistically significant difference compared to the other groups (*p* < 0.001). When the bacterial load levels between Groups IV and V were analysed, no statistically significant difference was found (*p* > 0.05). However, both groups had lower bacterial load levels compared to Group III. Consequently, the bacterial load in Group IV was the lowest among all groups and was statistically significant (*p* < 0.001).

### 2.5 Oxidative Stress Measurements

**FIGURE 2:**
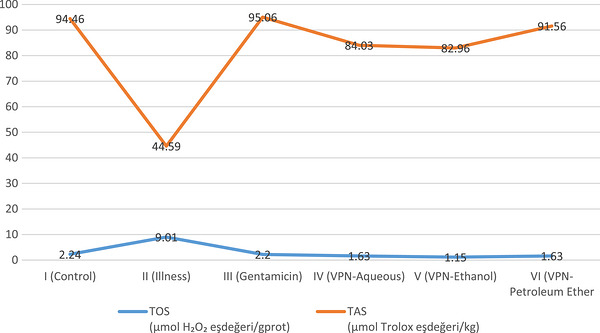
TOS levels were significantly elevated in the disease group (Group II) and decreased in all treatment groups (*p* < 0.000). No significant differences were found among the extract‐treated groups (*p* > 0.05). Similarly, TAS levels were lowest in Group II and significantly increased in all treatment groups (*p* < 0.000), with no significant difference among Groups IV, V and VI (*p* > 0.05). TOS, total oxidative status.; VPN, *Vitex pseudo‐negundo*.

The average TOS values (µmol H_2_O_2_ equivalent/g prot) were determined as follows: Group I (control): 2.24 ± 0.37, Group II (disease): 9.01 ± 1.06, Group III (gentamicin treated): 2.20 ± 0.90, Group IV (water extract treated): 1.63 ± 0.32, Group V (ethanol extract treated): 1.15 ± 0.30 and Group VI (petroleum ether extract treated): 2.98 ± 0.43, and the mean TAS values (µmol trolox equivalent/kg) were measured as follows: Group I (control): 94.46 ± 0.83, Group II (disease): 44.59 ± 1.58, Group III (gentamicin): 95.06 ± 2.31, Group IV (water extract treated): 84.03 ± 3.84, Group V (ethanol extract treated): 82.96 ± 2.21 and Group VI (petroleum ether extract treated): 91.56 ± 2.53 (Figure 2).

TOS levels in the disease group (Group II) were significantly higher compared to all other groups (*p* < 0.001). When evaluated among treatment groups, TOS levels in Group III (gentamicin‐treated group) were significantly lower compared to infected but untreated Group II (*p* < 0.001). TOS levels were significantly lower in the herbal extract‐treated groups (Groups IV, V and VI) compared to Group II (*p* < 0.001); however, there was no statistically significant difference in TOS levels between Groups IV, V and VI (*p* > 0.05).

TAS levels were significantly lower in Group II (disease) compared to all other groups (*p* < 0.001). Among the treated groups, TAS levels in Group III (gentamicin treated) were significantly higher compared to the disease group (*p* < 0.001). No statistically significant difference was found between the groups treated with herbal extracts (Groups IV, V and VI) in terms of TAS levels (*p* > 0.05), but TAS levels in these groups were significantly higher compared to Group II (*p* < 0.001).

### 2.6 Histopathological Examination

**FIGURE 3:**
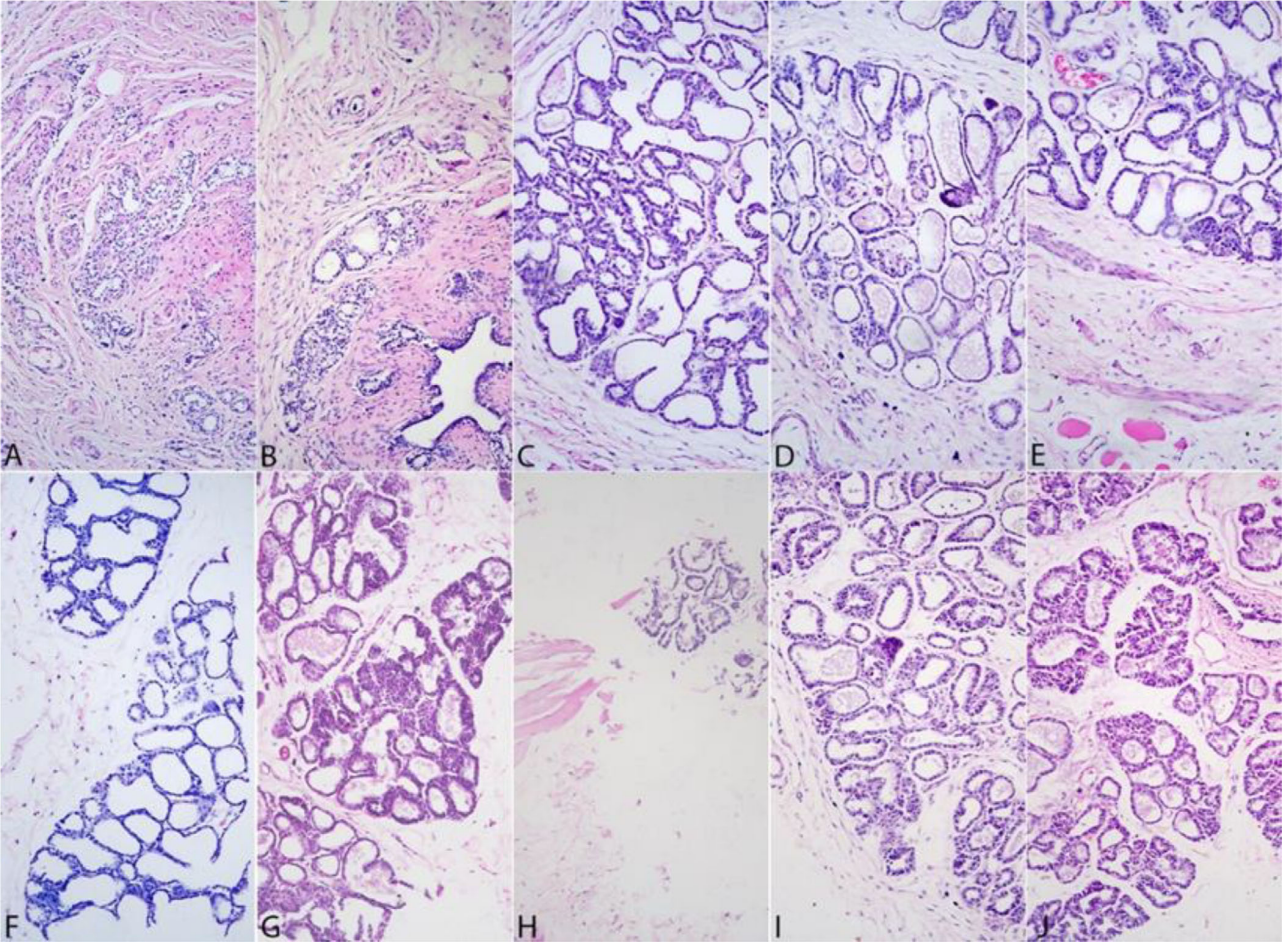
(A–J) Normal histologic appearance of mammary glands of different animals in Group I. H&E staining and ×20 objective magnification.

Microscopic examination revealed no pathological changes in the mammary tissues of Group I (Control), with normal glandular architecture and absence of inflammation (Figure 3).

**FIGURE 4:**
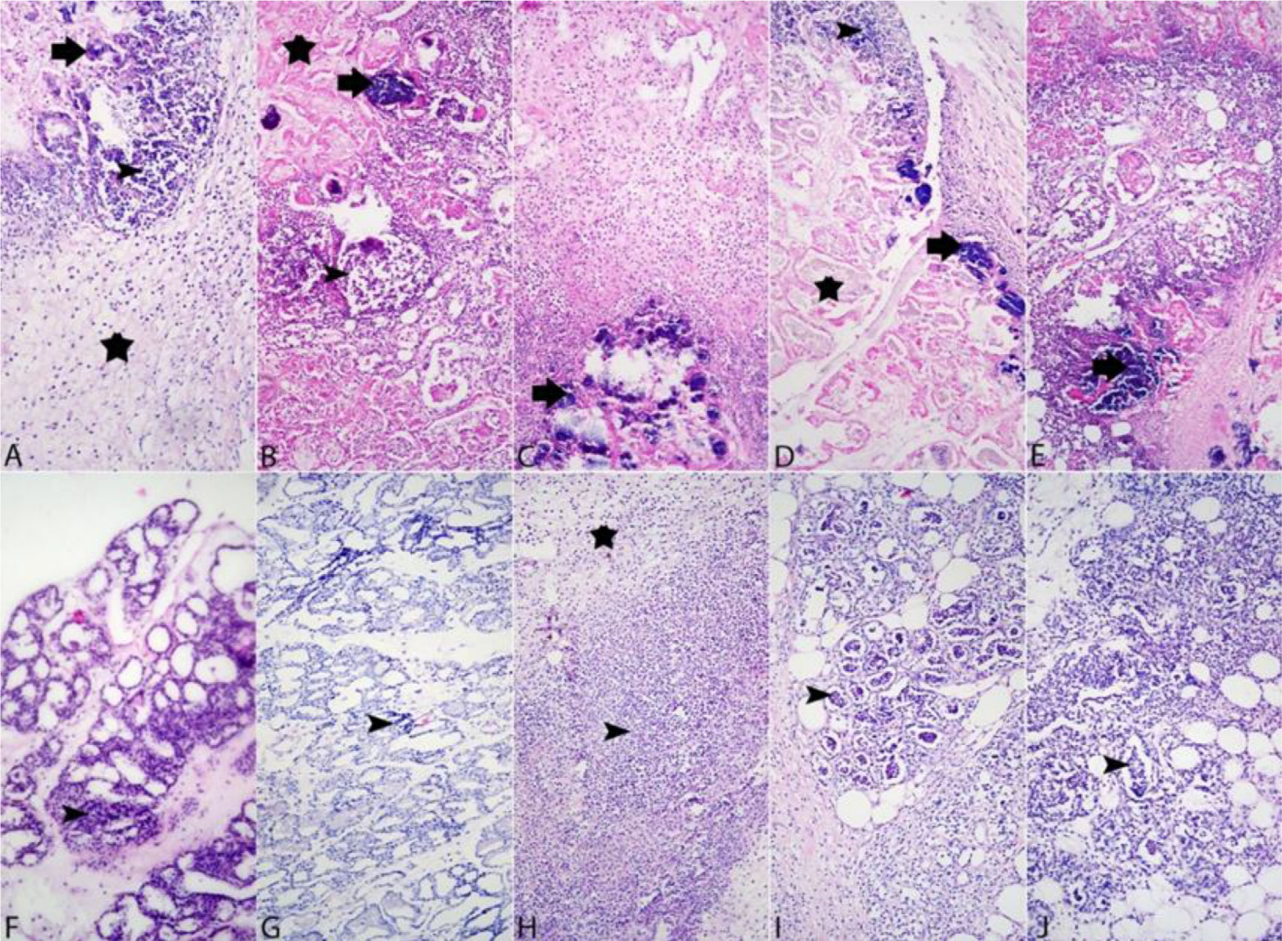
Histologic images of mammary gland of different animals in Group II. Numerous bacterial colonies (arrow) and dense inflammatory cell infiltration in the lumens and periphery of the mammary glands (arrowhead), dense necrosis in the mammary glands and interstitium (asterisk). ×20 objective magnification and H&E stain.

In Group II (disease), diffuse infiltration of plasma cells, lymphocytes, macrophages and neutrophils was observed, accompanied by significant degeneration, necrosis and multifocal bacterial clusters. The inflammatory profile was characterized by diffuse neutrophilic infiltration in the disease group, consistent with acute suppurative mastitis. In contrast, treatment groups showed a mixed population of macrophages, lymphocytes and plasma cells, indicating subacute to resolving inflammation and ongoing tissue repair. Severe fibrosis was noted, especially in samples G2‐8–G2‐10, whereas moderate inflammation and fibrosis were observed in G2‐6 and G2‐7 (Figure 4).

**FIGURE 5:**
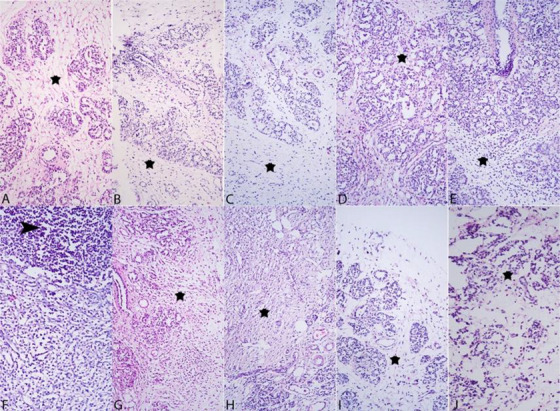
(A–J) Histologic images of mammary gland from different animals in Group III. In the interstitium, there is a marked increase in connective tissue (asterisk) and a decrease in inflammatory cell density. ×20 objective magnification and H&E staining.

Group III (gentamicin) showed localized pyogranulomatous lesions in several samples, with reduced inflammation and preserved glandular structures compared to Group II. Some samples (e.g., G3‐7) demonstrated complete resolution of inflammation, whereas others exhibited moderate fibrosis (Figure 5).

**FIGURE 6:**
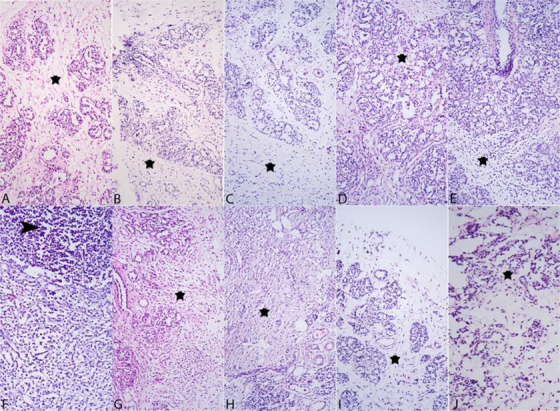
(A–J) Histopathologic images of mammary glands from different animals in Group VI. Dense connective tissues increase in the interstitium (asterisk) and focal inflammatory cell infiltration (arrowhead). ×20 objective magnification and H&E staining.

In Group IV (water extract), significant degeneration and necrosis were observed in early samples (e.g., G4‐1), whereas others (G4‐2 and G4‐3) showed complete regression of inflammation but persistent fibrosis. Focal pyogranulomatous lesions and epithelial degeneration were noted in some samples, with varying degrees of fibrosis (Figure 6).

**FIGURE 7:**
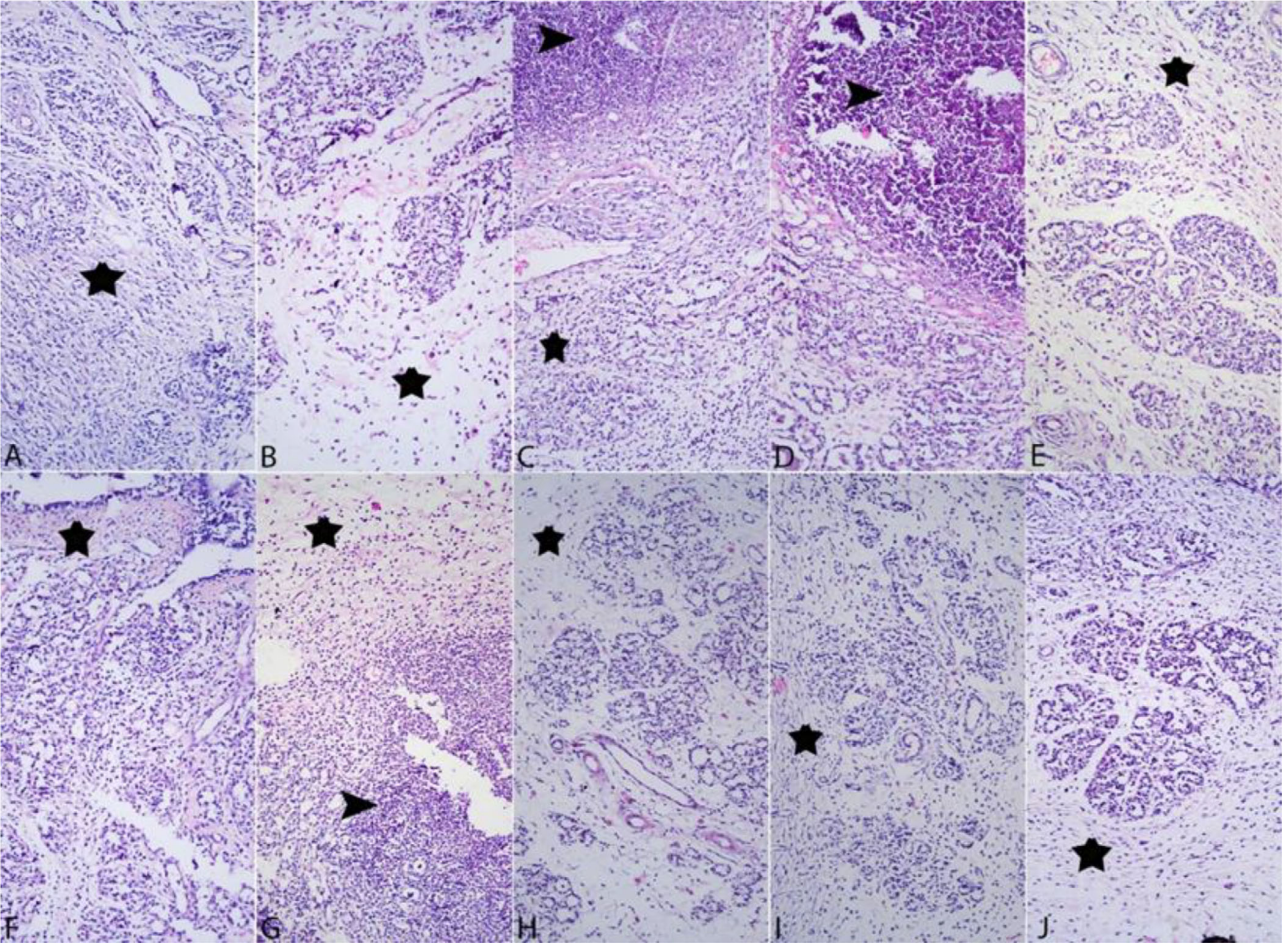
(A–J) Histologic images of mammary gland from different animals in Group V. Prominent connective tissues increase in the interstitium (asterisk) and focal inflammatory cell infiltration (arrowhead). ×20 objective magnification and H&E staining.

Group V (ethanol extract) displayed moderate inflammatory infiltration with mixed cell populations and moderate glandular deterioration. Some samples (e.g., G5‐8) exhibited minimal inflammation and well preserved glandular structures, whereas others had significant connective tissue proliferation (Figure 7).

**FIGURE 8:**
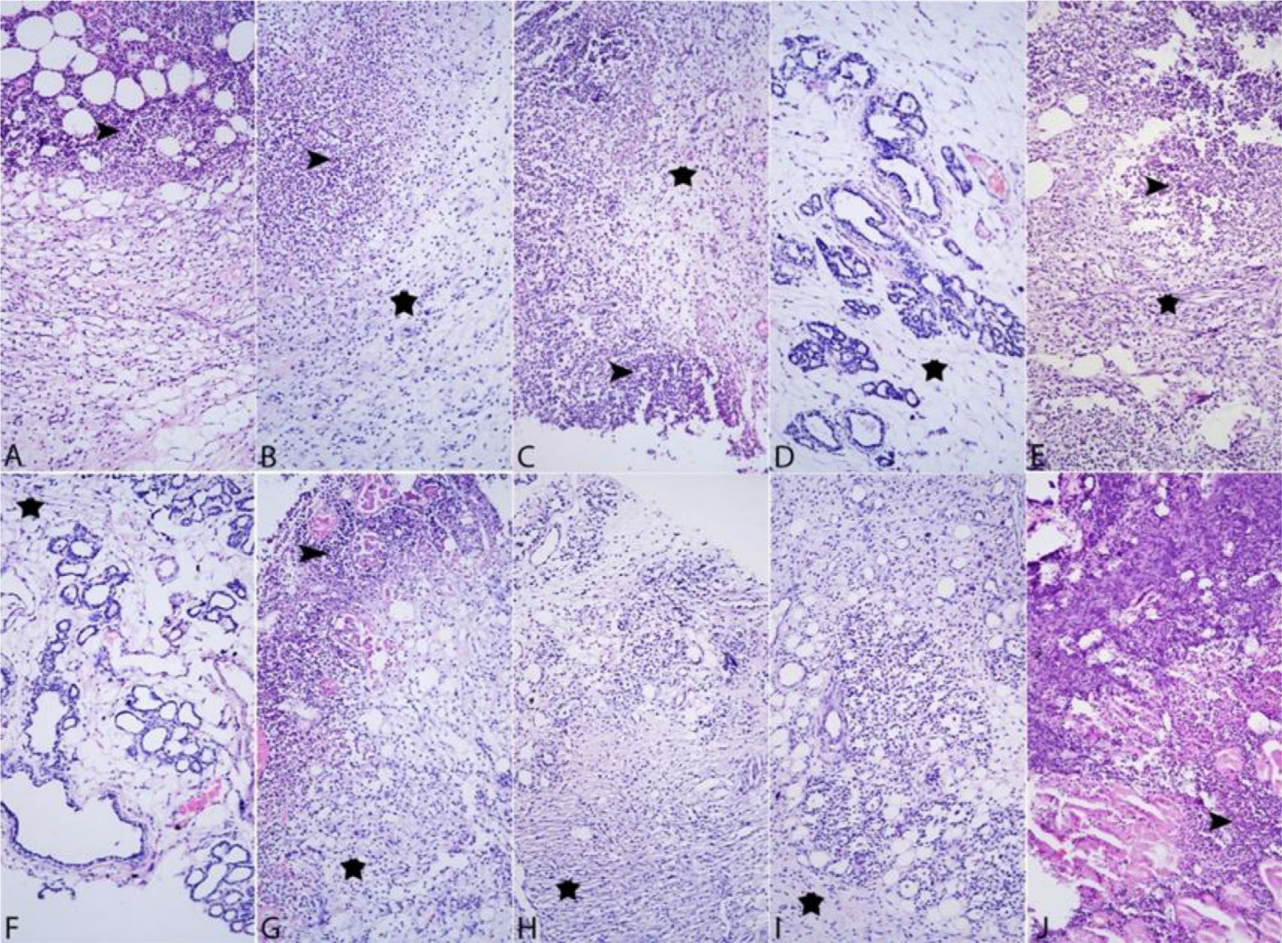
(A–J) Histologic images of mammary glands from different animals in Group VI. Prominent connective tissues increase in the interstitium (asterisk) and inflammatory cell infiltration (arrowhead). ×20 objective magnification and H&E staining.

In Group VI (petroleum ether extract), diffuse infiltration of mononuclear and neutrophil cells was observed. Although mild to severe glandular degeneration was present, several glands retained normal architecture. Mild inflammation and slight connective tissue proliferation were noted in G6‐4 and G6‐6 (Figure 8).

Summary (±SD) of mean experimental outcomes between groups for key parameters such as mammary gland weights, bacterial load, TOS and TAS and histopathological scoring for VPN extracts and gentamicin on mastitis outcomes in rats (Table 1). Histopathological lesions were semi‐quantitatively scored (0–3 scale) and summarized in Table 1, demonstrating significantly higher scores in the disease group compared to treatment and control groups (*p* < 0.001).

**TABLE 1:** Summary of mean experimental results across groups (±SD) for key parameters.

Group	Treatment	Mammary gland weight (g)	Bacterial load (log_10_ cfu/g)	TOS (µmol H_2_O_2_ equivalent/gprot)	TAS (µmol trolox equivalent/kg)	Histopathology score^a^	Clinical recovery day
I	Healthy control	1.10 ± 0.03	5.40 ± 0.10	2.24 ± 0.37	94.46 ± 0.83	0	—
II	Infected (untreated)	2.11 ± 0.04	9.37 ± 0.06	9.01 ± 1.06	44.59 ± 1.58	3	No recovery
III	Gentamicin	1.51 ± 0.03	6.83 ± 0.15	2.20 ± 0.90	95.06 ± 2.31	1	Day 3
IV	VPN‐(water)	1.58 ± 0.01	4.34 ± 0.09	1.63 ± 0.32	84.03 ± 3.84	1	Day 5
V	VPN‐(ethanol)	1.61 ± 0.02	4.66 ± 0.16	1.15 ± 0.30	82.96 ± 2.21	2	Day 5
VI	VPN‐(petroleum ether)	1.60 ± 0.04	5.24 ± 0.31	1.63 ± 0.37	91.56 ± 2.53	1	Day 5

Abbreviations: TOS, total oxidative status; VPN, *Vitex pseudo‐negundo*.

^a^ Histopathology score based on severity of inflammation and tissue damage: 0: none, 1: mild, 2: moderate and 3: severe.

**FIGURE 9:**
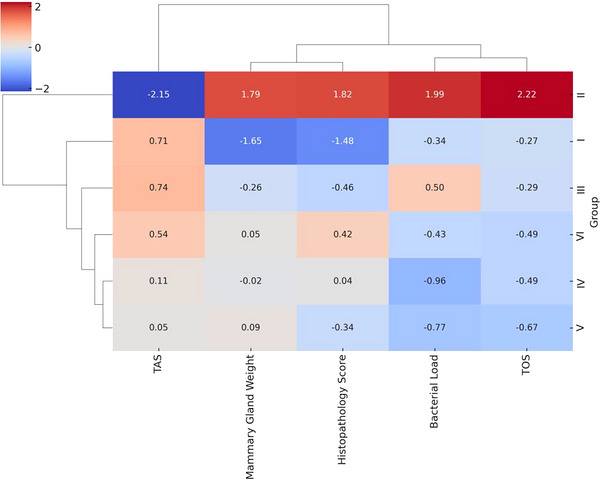
Clustered image map (CIM) displaying the similarity between experimental groups and key biological variables. TOS, total oxidative status.

Clustered image map summarizing how treatment groups and measured variables are hierarchically grouped, highlighting treatment effects (Figure 9).

The clustered image map provided a comprehensive visualization of the experimental groups based on standardized biological parameters. Clear clustering patterns emerged, highlighting differences in physiological and biochemical responses. The infected untreated group (Group II) clustered independently, exhibiting high values for TOS, bacterial load and histopathology score, whereas it showed markedly reduced TAS, indicative of oxidative damage and severe inflammation. In contrast, VPN‐treated groups (IV–VI) and gentamicin group (III) formed a distinct cluster, characterized by improved antioxidant levels, reduced bacterial burden and lower tissue damage scores. Among treatments, the VPN–ethanol and VPN–water extracts demonstrated clustering proximity to gentamicin, suggesting comparable biological activity. The healthy control (Group I) clustered separately, consistent with baseline physiological conditions. Overall, the clustering patterns validate the therapeutic potential of VPN extracts, particularly in reducing oxidative and infectious burden, and support their effectiveness as alternative or adjunct therapies to conventional antibiotics.

**FIGURE 10:**
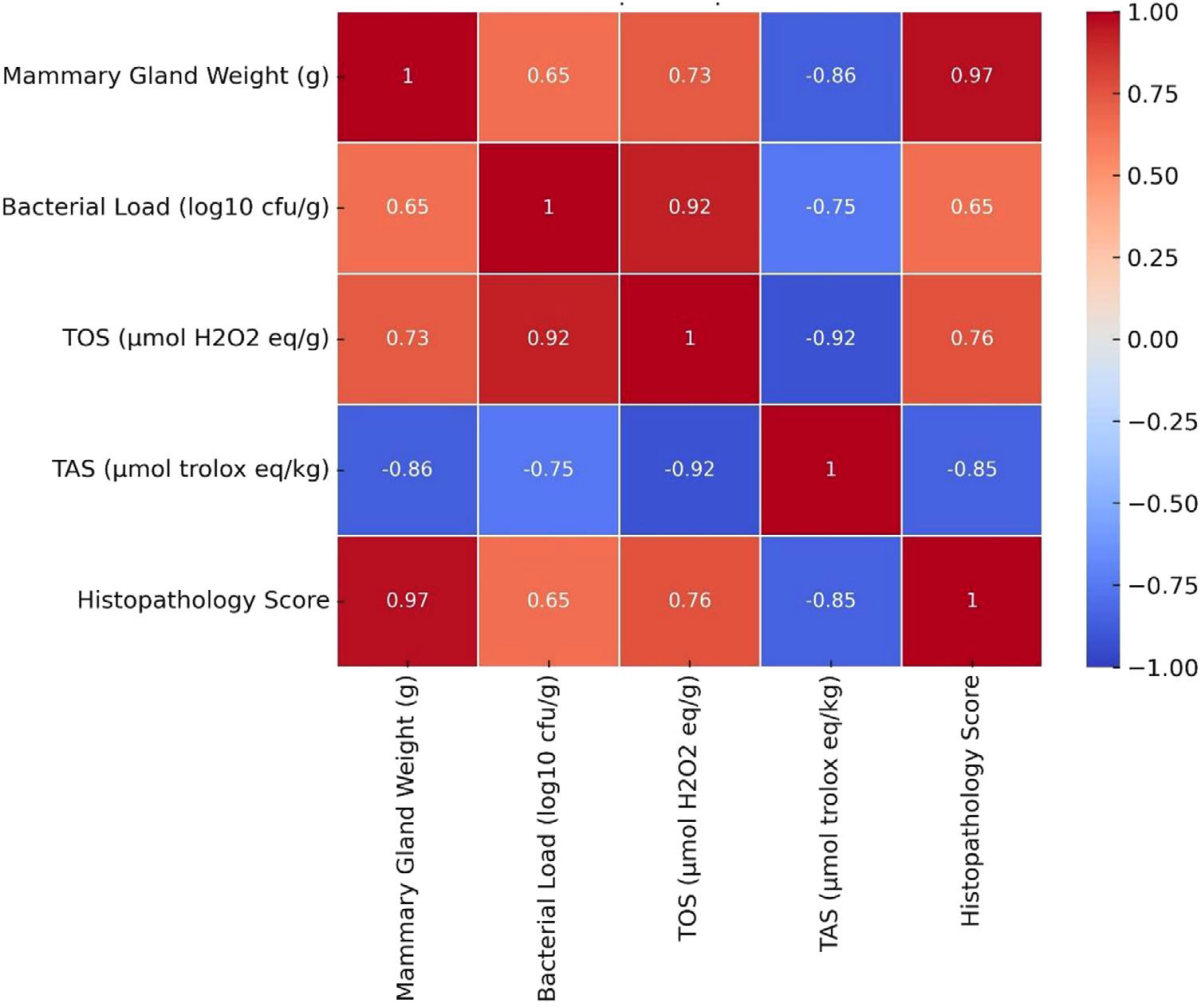
Correlation heat map depicting relationships among oxidative stress, bacterial load, antioxidant capacity and tissue pathology. TOS, total oxidative status.

The correlation heat map showing the relationships between clinical, biochemical, bacteriological and histopathological variables shows the main relationships (Figure 10).

The correlation heat map revealed significant relationships among key experimental parameters. A strong positive correlation was observed between histopathology score and both mammary gland weight (*r* = 0.97) and TOS levels (*r* = 0.76), indicating that increased tissue damage and inflammation are associated with oxidative stress and tissue swelling. Similarly, bacterial load correlated strongly with TOS (*r* = 0.92), reinforcing the role of infection‐induced oxidative damage. Conversely, TAS (total antioxidant status) showed a strong negative correlation with TOS (*r* = −0.92), histopathology (*r* = −0.85) and mammary weight (*r* = −0.86), suggesting that antioxidant defence mechanisms are suppressed under severe inflammation and oxidative conditions. Overall, the correlation profile supports the hypothesis that the therapeutic efficacy of treatments is closely linked to their capacity to reduce oxidative stress, bacterial burden and tissue injury.

**FIGURE 11:**
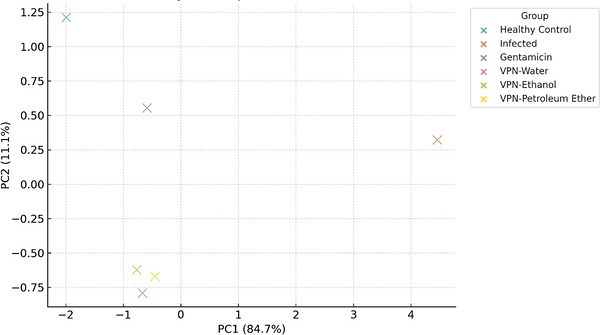
Principal component analysis (PCA) showing the distribution of experimental groups based on standardized biological parameters. VPN, *Vitex pseudo‐negundo*.

Principal component analysis (PCA) showing the distribution of experimental groups based on standardized biological parameters (Figure 11).

The PCA plot demonstrates clear separation among experimental groups based on the variation in oxidative, microbiological and histopathological parameters. PC1, accounting for 84.7% of the total variance, primarily distinguishes the infected untreated group (Group II) from all others, indicating a distinct pathophysiological profile characterized by elevated bacterial load, oxidative stress and tissue damage. On the opposite side of the PCA space, VPN‐treated groups (IV–VI) cluster more closely, suggesting similar biological responses and therapeutic effects. Notably, VPN–ethanol and VPN–water groups show proximity to the gentamicin group (III), implying comparable efficacy in modulating key parameters. The healthy control (Group I) is well‐separated, reinforcing its baseline status. These findings highlight the discriminative power of PCA in visualizing the impact of different treatments and support the therapeutic relevance of VPN extracts.

## 3 Discussion

Mastitis is characterized by inflammation and swelling of the mammary tissue, leading to significant economic losses in dairy cattle. The experimental rat mastitis model demonstrates that infection‐induced inflammation leads to a marked increase in mammary gland weight.

Here, mammary gland weight was significantly higher in the infected untreated group compared with all other groups, reflecting inflammation‐associated oedema in the mammary tissue. These findings are consistent with the known pathophysiological consequences with mastitis. The markedly increased mammary gland weight in the illness group specifically suggests that the inflammatory response induced by SA leads to tissue oedema, which is a crucial factor in the progression of the infection. Previous studies similarly report that mastitis is associated with increased mammary gland weight due to inflammatory swelling. Already stated earlier, literature indicates that mastitis induces harmful tissue alterations by elevating mammary gland weight (Taifa et al. 2022).

Gland weight was significantly reduced in the gentamicin‐treated group compared with the infected untreated group (*p* < 0.001?), indicating effective mitigation of inflammation and oedema (Taifa et al. 2022). Copper nanoparticles (CuNPs) have shown similar therapeutic effects in mastitis models (Taifa et al. 2022). Groups treated with herbal extract also exhibited significantly lower gland weights than the diseased group (*p* < 0.001?), and their values did not differ significantly from the gentamicin group (*p* > 0.05), suggesting comparable anti‐inflammatory activity. Prior studies support the antibacterial and antioxidant properties of VPN (Almuslih and Al‐Assie 2020), which may contribute to reductions in oxidative stress and inflammation. Natural antioxidants, such as nanocurcumin, have demonstrated similar therapeutic effects (Suresh et al. 2022). Together, these findings indicate that both antibiotic and herbal therapies are capable of reducing inflammation and oxidative damage associated with mastitis. Further studies are needed to identify the active constituents of the extracts and elevate their long‐term therapeutic effects.

The result of this study indicates that mastitis markedly increases mammary gland weight and that the tested herbal extracts exhibit anti‐inflammatory effects comparable to gentamicin. Additional research on plant‐based? (did you mean that?) may help advance the development of alternative mastitis therapies. CRP levels remained below 0.3 mg/dL in all groups, suggesting the absence of a significant systemic inflammatory response. Although CRP typically increases during systemic inflammation, the consistently low levels observed in this study indicate that the mastitis model triggered a primarily localized inflammatory response (Aydın and Emre 2021). The diagnostic utility of CRP in mastitis remains debated, with some studies reporting limited specificity (Douglas 2023). Therefore, mastitis evaluation may benefit the combined use of both local and systemic inflammatory markers.

Mastitis is a prevalent udder disease in dairy livestock, leading you to reduced milk production and economic losses. Although antibiotics are widely used, concerns related to such resistance and drug residues highlight the need for alternative therapies (Haghighi and Saharkhiz 2021). This study explored the antibacterial efficacy of VPN extracts. Significant differences in bacterial load were observed between the disease group (Group II) and treated groups. Group IV exhibited the lowest bacterial burden (4.34 ± 0.09 log_10_ cfu/g), supporting the strong antibacterial potential of the aqueous extract (Almuslih and Al‐Assie 2020; Díaz et al. 2003; Haghighi and Saharkhiz 2021).

Mastitis induced by SA led to an increased bacterial load and mammary gland weight, reflecting severe inflammation (Taifa et al. 2022). Both gentamicin and VPN extracts (Groups IV–VI) significantly reduced bacterial burden and tissue oedema. No significant difference was observed between Groups IV and V (*p* > 0.05), indicating comparable antibacterial efficacy. The aqueous extract demonstrated the greatest antibacterial activity, possibly due to better solubility of its active constituents. In contrast, the petroleum ether extract (Group VI) showed relatively limited antibacterial activity, which may be related to reduced solubility of its bioactive components (Haghighi and Saharkhiz 2021).

Although nanotechnological treatments, such as CuNPs, have shown promise (Taifa et al. 2022), herbal extracts may offer advantages due to their lower toxicity and greater sustainability (Díaz et al. 2003). Overall, aqueous VPN extract demonstrated the strongest antibacterial effects, underscoring the potential of herbal therapies as alternatives to conventional antibiotics. Further studies using larger models and mechanistic analyses are warranted.

This study also evaluated oxidative stress markers, TOS and TAS, in the rat mastitis model. The results showed that mastitis significantly increased TOS and decreased TAS compared with the control group (*p* < 0.001), indicating heightened oxidative stress and reduced antioxidant capacity. Inflammation‐associated with mastitis promotes excessive production of reactive oxygen species (ROS), which damage lipids, proteins and DNA, ultimately leading to cellular dysfunction (Novac and Andrei 2020; Özcan et al. 2015). Similar oxidative imbalances have been reported in cows with SA‐induced mastitis (Kurt et al. 2021).

Gentamicin treatment significantly reduced TOS and increased TAS compared with the diseased group (*p* < 0.001), likely as a result of reducing bacterial burden and inflammation. Similarly, VPN extracts (groups IV, V and VI) significantly improved oxidative balance (*p* < 0.001), supporting their therapeutic potential. No statistically significant differences were observed among extracts (*p* > 0.05), indicating comparable antioxidant effects under the tested conditions. Previous studies have also documented the antioxidant properties of VPN supporting its potential role in mitigating oxidative damage (Almuslih and Al‐Assie 2020; Kadir et al. 2013; Seresht et al. 2021). However, solvent type can influence extract bioactivity, as methanolic preparations have demonstrated increased antioxidant and anticancer activity in previous studies (Shiri Aghbash et al. 2024). Overall, mastitis increased oxidative stress and suppressed antioxidant capacity, whereas VPN extracts effectively reversed these alterations, supporting their potential as adjunct treatments for mastitis.

Mastitis induces pronounced histopathological changes in the mammary gland.

In this study, SA induced mastitis resulted in extensive neutrophilic infiltration, bacterial colonies, tissue degeneration and fibrosis. Histopathological assessment revealed diffuse infiltration of neutrophils, lymphocytes, plasma cells and macrophages, accompanied by connective tissue proliferation, indicating active inflammatory and reparative processes (Gogoi‐Tiwari et al. 2015; Suresh et al. 2022; Taifa et al. 2022). Gentamicin treatment (Group III) significantly reduced inflammation and helped preserve glandular structure, although fibrosis remained as part of repair process. Similar outcomes have been reported with nanotechnology‐based treatments, in which inflammation decreases but residual fibrotic tissue remains (Taifa et al. 2022). The herbal extract‐treated groups (IV, V and VI) also showed diminished inflammation and tissue degeneration. The water and ethanol extracts of VPN were particularly effective in reducing inflammation and limiting connective tissue proliferation. These effects may be related to the anti‐inflammatory constituents of VPN, such as flavonoids and phenolic compounds, which are known to modulate immune responses (Almuslih and Al‐Assie 2020; Díaz et al. 2003; Haghighi and Saharkhiz 2021). In Group VI (petroleum ether extract), inflammation regresses only slightly, suggesting lower bioavailability or reduced concentrations of active compounds. Overall, both antibiotic and herbal treatments substantially reduced the severe degeneration and necrosis observed in the disease group, reinforcing their therapeutic potential. Persistent fibrosis across treatment groups reflects ongoing tissue repair but may also carry of a risk lasting mammary tissue alteration if unresolved (Suresh et al. 2022; Taifa et al. 2022). In summary, the water and ethanol extracts of VPN demonstrated strong anti‐inflammatory effects comparable to antibiotics, offering their potential as herbal alternatives for mastitis treatment. Future studies should focus on optimizating dosage and identifying active compounds, and elucidating underlying molecular mechanism to enhance therapeutic applicability.

Although in vitro cytotoxicity assays are commonly used in early pharmacological screening, the present study was primarily designed to evaluate the in vivo therapeutic efficacy of VPN extracts in an experimental mastitis model (Sindhu et al. 2025). During the experimental period, no clinical signs of systemic toxicity were observed in any treatment group. Furthermore, histopathological examination did not reveal any extract‐related cellular degeneration or necrosis in mammary tissues, suggesting that the administered doses were well tolerated in vivo (Mahfuz‐Al‐Mamun et al. 2015). The safety of plant‐derived extracts is frequently assessed in vivo following initial antimicrobial screening, as several studies have demonstrated that many phytotherapeutic agents exhibit low cytotoxicity while maintaining antibacterial activity against mastitis pathogens (Arbab et al. 2022; Erhabor et al. 2025). Previous studies have also reported that many plant‐derived compounds used in mastitis research exhibit significant antibacterial and anti‐inflammatory properties while demonstrating relatively low cytotoxicity (Hashem et al. 2024; Srichok et al. 2022; Sriyanti et al. 2023; Suresh et al. 2022; Taifa et al. 2022).

The use of SA as the experimental pathogen was based on its major epidemiological importance in bovine mastitis. Numerous studies have demonstrated that SA is one of the most prevalent etiological agents of intramammary infections and is frequently associated with chronic and recurrent mastitis due to its ability to form biofilms and evade host immune responses. Although bovine mastitis has a multifactorial aetiology involving several bacterial species, the use of a single well‐characterized pathogen, such as SA, enables the development of standardized experimental models, facilitating the evaluation of therapeutic strategies and host–pathogen interactions under controlled laboratory conditions (Gogoi‐Tiwari et al. 2017; Peton et al. 2014; Raza et al. 2013; Wang et al. 2022).

## 4 Conclusion

This study demonstrated that water and ethanol extracts of VPN significantly improved clinical symptoms, reduced bacterial load and decreased oxidative stress in an experimental rat mastitis model, showing effectiveness comparable to gentamicin. Histopathological improvements were more pronounced in the group treated with water extract cohort. These findings suggest that VPN extracts may serve as effective alternative or adjunct therapies mastitis. Further studies using larger animal models and molecular analyses are needed to confirm safety, long‐term efficacy and underlying mechanisms of action.

## Author Contributions

Hüseyin Koç conceived the study idea, established the experimental animals, performed the biochemical analysis, collected, dried and ground the plants from the field and organized and statistically analysed the data. Arzu Findik performed and evaluated the microbiological analysis. Tolga Güvenç conducted and evaluated the histopathological analyses. Nevzat Batan participated in fieldwork and plant identification. Seda Fandakli performed the extraction process. Murat Findik contributed to the study design, participated in the scientific planning and assisted with the animal model.

## Funding

This study was supported by TUBITAK 1002 Project, Türkiye (Project number: 1002‐124O594).

## Ethics Statement

The study received ethical approval from the Karadeniz Technical University Animal Experiments Local Ethics Committee (Decision Date: 13 June 2024; Decision Number: 2024/10 and Number 53488718‐352). The authors confirm that the ethical policies of the journal, as noted on the journal's author guidelines page, have been adhered to.

## Conflicts of Interest

The authors declare no conflicts of interest.

## Data Availability

The data supporting the findings of this study are available from the corresponding author upon reasonable request.
